# The reciprocal effects of physical activity and happiness in adolescents

**DOI:** 10.1186/s12966-020-01058-8

**Published:** 2020-11-19

**Authors:** Thabo J. van Woudenberg, Kirsten E. Bevelander, William J. Burk, Moniek Buijzen

**Affiliations:** 1 Erasmus School of Social and Behavioural Sciences, Erasmus University Rotterdam, Burgemeester Oudlaan 50, 3062 PA Rotterdam, the Netherlands; 2 Behavioural Science Institute, Radboud University, Montessorilaan 3, 6525 HR Nijmegen, the Netherlands; 3 Radboud Institute for Health Sciences, Primary and Community Care, Radboud University and Medical Center, Geert Grooteplein Noord 21, 6525 EZ Nijmegen, the Netherlands

**Keywords:** Physical activity, Happiness, Well-being, Adolescents

## Abstract

**Background:**

Positive associations exist between physical activity and happiness in adolescents. However, previous studies have mostly used self-reported measures and cross-sectional designs. There is a need for more insight into the directionality and duration of this association. The current study was the first to investigate whether an increase in physical activity leads to happiness and whether adolescents become more physically active when they are happier. These two effects were studied between (on a day-to-day basis) and within days (on an hour-to-hour basis).

**Methods:**

The study used data from the *MyMovez* project in which 1484 adolescents between the ages of 8 and 17 years wore an accelerometer on their wrist and answered experience sampling questions on happiness at random moments during the day for several weeks in 2016–2018.

**Results:**

The preregistered analyses demonstrated an association between physical activity and happiness. More specifically, the number of steps per day predicted the experienced happiness on that day. In addition, a short-term reciprocal effect of physical activity and happiness was observed. Happiness was predicted by the number of steps accumulated in the previous hour and it also predicted the number of steps accumulated in the subsequent hour. However, convincing evidence was found that these effects did not occur in the long-term between days. The number of steps on the previous day did not predict happiness, nor did happiness predict the number of steps of the subsequent day.

**Conclusions:**

This study confirms an association between physical activity and happiness in adolescents and shows that in the short-term, physical activity promotes happiness and vice versa. Therefore, we conclude that physical activity is not only important for the physical health of youth, but also plays an important role in their mental well-being. In addition, this knowledge can be used to further understand the importance of physical activity in adolescents’ health and help in promoting a healthy lifestyle among youth.

**Trial registration:**

The data used are stored at the Data Archiving and Networked Services (10.17026/dans-zz9-gn44). Hypotheses, study design, sample, data collection procedure, measured variables, and plan of analysis were preregistered on the Open Science Framework (OSF, https://osf.io/5yk7r/).

## Introduction

In the past decades, subjective well-being in adolescents has become a major topic in public policy [1]. Low subjective well-being has serious consequences on adolescents’ lives, as this can lead to behavioral and social problems, lowered self-esteem and academic achievement, and depression [2]. Subjective well-being is a complex construct that is defined and measured in countless ways [3], but scholars often make a distinction between the individual’s overall evaluation of life and an individuals’ affective state, also referred to as happiness [4]. In this study, we focus on happiness as the experience of positive feelings throughout the day (*affective* or *hedonic* well-being) as opposed to general life satisfaction and sense of purpose in life (*eudaemonic* and *evaluative* well-being) [1, 4, 5]. Besides happiness being regarded as one of the most fundamental goals in life [6], increased happiness is also suggested to be an important precursor of adolescents’ general health [1]. Therefore, it is important to understand what promotes and increases happiness in adolescents.

Physical activity is generally presumed to be associated with experiences of happiness in adolescents [7, 8]. An extensive set of studies has shown that there is a positive association between physical activity and happiness in the wider population, including adolescents (for a review see: [9]). More specifically, previous cross-sectional studies have demonstrated a positive association between self-reported physical activity and mental well-being [10, 11] or happiness in adolescents [12–14]. In these studies, adolescents reported higher levels of happiness when they reported a higher number of days on which they met the norm for physical activity (> 60 min of moderate to vigorous physical activity per day) or when they reported more days of being part of after-school physical activities.

Two complementary mechanisms clarify how physical activity and happiness are related to each other. Interestingly, they propose opposing directions in the association between the two concepts. On the one hand, a directional effect of physical activity on happiness can be explained by physiological processes. Physical activity enhances the transmission of monoamines (i.e., noradrenaline, dopamine, and serotonin) in the brain [15] and increases the production of endorphins [16]. Both physiological responses are known to decrease depressive symptoms and anxiety and to promote well-being [17]. On the other hand, the effect of happiness on physical activity can be explained by cognitive and affective processes, as described in the theory of planned behavior [18], self-determination theory [19, 20], the trans-theoretical model [21], and affective models of behavior change [22]. More specifically, positive affect, such as happiness, influences the intentions to engage in physical activity [23], and anticipated affective experiences predict future physical activity behavior [22]. Moreover, negative affect in particular has a detrimental effect on the intentions to be physically active [23].

Notwithstanding the extensive work that has been done on this topic, the previously conducted studies were unable to determine which of these two mechanisms explains how physical activity and happiness are related [8]. That is, these studies have not been able to infer the direction of the relationship between physical activity and happiness because of two important reasons. First, the aforementioned studies have used retrospective self-reported measures. Retrospective and self-reported data have been criticized for several shortcomings [1]. For example, asking adolescents to report on their feelings and behaviors retrospectively might lead to memory bias, socially desirable answers, or being affected by primacy and recency effects [24]. Also, by asking adolescents to report on both physical activity and happiness in the same questionnaire, associations are likely to be inflated due to shared method variance and are prone to response set biases [25]. To address these biases, the current study used accelerometers to continuously measure physical activity and an experience sampling methodology (ESM) to measure happiness at random moments during the day [1, 24].

Second, the aforementioned studies were not designed to test the directionality of the association between physical activity and happiness as they used cross-sectional designs or lacked repeated measures of either physical activity or happiness [9, 26, 27]. To determine the directionality, longitudinal (or repeated) observations are needed [28]. As far as we know, only one study used a longitudinal design to investigate the association between physical activity and well-being (a combination of *eudaemonic*- and *hedonic* well-being) in adolescents [29]. Unfortunately, this study only measured physical activity (by using accelerometers) at baseline, whereas the measure of mental well-being was 3 years later. The results showed no evidence of an association between physical activity (both volume and intensity) and mental well-being 3 years later. The authors acknowledged that the design could be improved by including repeated measures of physical activity and mental well-being over time [29], and they stress that there is a need to establish the directionality of this effect in order to better understand how physical activity can be used to improve mental health and vice versa [29, 30].

## The current study

The objective of the current study was to further examine the association between physical activity and happiness in adolescents. The first aim was to test the association between physical activity and happiness in adolescents on a day to day level. The study tested whether happiness measured at a random moment during the day was predicted by the number of steps that was accumulated on that day in a mixed-effects framework. Based on the generally observed correlation between self-reported physical activity and happiness in previous studies, we hypothesized that (H1a) the daily number of steps would positively predict happiness in adolescents.

The detailed level of the data (i.e., multiple measurements per participant) allowed both the between- and within-subjects effects of physical activity on happiness to be investigated. This means that we could determine whether having a physically active lifestyle or having a relatively active day contributes more to happiness in adolescents. We anticipated that (H1b) the average number of steps per participant would positively predict happiness in adolescents. In addition, we were also interested in uncovering whether relative increases in the participants’ average physical activity led to increases in their experiences of happiness. Therefore, we looked at the deviations in the average number of steps per participant to determine relative changes in physical activity and anticipated that (H1c) the relative increase in the number of steps per day per participant would positively predict happiness in adolescents.

The second aim of this study was to investigate the direction of the association between physical activity and happiness. So far, it has remained unclear what the most appropriate time interval is for examining this association. Urged by a recent review to “test for different time periods between physical activity and future effects on mental health” [8], we preregistered two different time frames in the current study: a day-to-day time frame and an hour-to-hour time frame.

**Fig. 1:**
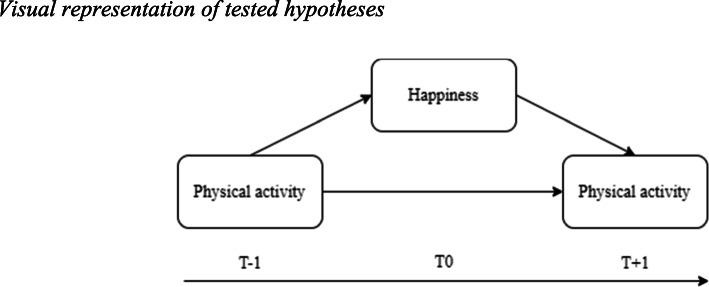
Visual representation of tested hypotheses

First, the effect of the physical activity of the previous day (T-1) on happiness and the effect of happiness on the physical activity of the subsequent day (T + 1) were investigated. Based on the physiological and the psychological explanations of the associations between physical activity and happiness, we hypothesized that (H2a) happiness in adolescents would be positively predicted by the number of steps on the previous day and that (H2b) the number of steps of the subsequent day would be positively predicted by happiness in adolescents. A potential confounding factor of the effect of happiness on physical activity is that the number of steps on the previous day predicts both happiness and the number of steps of the subsequent day. In order to isolate the indirect effect of happiness on physical activity, while controlling for the autoregressive effect of physical activity, we used a mediation model in which the number of steps on the previous day predicted the number of steps on the subsequent day, mediated by happiness. Figure 1 gives a visual representation of the tested hypotheses. In this model, we anticipated that (H2c) the autoregressive effect of the number of steps on the previous day on the number of steps on a subsequent day would be partially mediated by happiness in adolescents.

Second, the same approach was used to test whether the reciprocal effects occurred in the time frame of an hour. More specifically, we anticipated that (H3a) happiness in adolescents would be positively predicted by the number of steps in the previous hour, (H3b) the number of steps of the subsequent hour would be positively predicted by happiness in adolescents, and that (H3c) the autoregressive effect of the number of steps in the previous hour on the number of steps in the subsequent hour would be partially mediated by happiness in adolescents. Due to the experience sampling procedure, the autoregressive effect of happiness could not reliably be assessed because of the varying time intervals between two subsequent observations of the participant’s happiness.

## Methods

### Participants and procedure

The study used data from the *MyMovez* project [31] which are stored at Data Archiving and Networked Services in the Netherlands (10.17026/dans-zz9-gn44). The project used a longitudinal design and investigated healthy lifestyles of adolescents in the Netherlands. In the project, 17 primary school and 11 secondary school classes participated in one or more of the seven data collection waves spread over 3 years (for an overview of the measurement periods, see Table 1). Participants received the *MyMovez* Wearable Lab for seven consecutive days, which consisted of a research smartphone with the corresponding *MyMovez* app and a wrist-worn accelerometer. On the smartphone, participants randomly received daily questionnaires (between 7:00 h and 19:30 h). To make participating more fun, participants were allowed to play a game for 5 min per hour and could chat with each other on the social media platform ‘Social Buzz’ during the last three waves of the project. The accelerometer was used to measure the amount and intensity of physical activity per minute.

**Table 1:** Overview of the measurement periods

Wave	Number of Participants	Start	Finish
1	843	27-01-2016	09-03-2016
2	901	31-03-2016	17-05-2016
3	868	01-06-2016	22-06-2016
4	744	15-02-2017	28-03-2017
5	1017	02-02-2018	17-04-2018
6	755	13-04-2018	06-06-2018
7	745	16-05-2018	04-07-2018

*Note. Start* is the first day of the first participants and *Finish* is the last day of the last participants

In these 3 years, a total of 1484 individual adolescents participated in 130 classrooms. The participants were between 8 and 17 years old (*M*_Age_ = 11.23, *SD*_Age_ = 1.74, 46.11% male). For an overview of the number of participants and dates per wave see Table 1. The hypotheses, study design, sample, data collection procedure, measured variables, and plan of analysis were preregistered on the Open Science Framework (OSF, https://osf.io/5yk7r). A subset of the data (wave 4) was used to determine the syntax for the analyses and was excluded from the analysis presented in this paper.

### Measures

#### Physical activity

Participants wore the accelerometer (Fitbit Flex™) on their non-dominant wrist for the length of the measurement period. Per day, data were gathered between 7:00 h and 19:30 h. Incomplete days of data (< 750 min of accelerometer data) were excluded from the data. Potential reasons for partial data were that the materials were distributed or collected at the schools, empty batteries, or non-wear time of the accelerometer [32]. Therefore, the maximum number of physical activity data was 5 days per wave. The amount of physical activity was expressed by the number of steps per day (or per minute). The average physical activity of the participants ranged between 1163.00 and 21,032.50 steps per day (*M*_Steps_ = 8931.15, *SD*_Steps_ = 2941.81, *Mdn*_Steps_ 8570.18). In addition, the variance within the participant’s physical activity was assessed. On average, participants deviated 3042 (*SD =* 2.593) steps per day from their mean number of steps.

Subsequently, the number of steps per day per participant were averaged to create a variable for the mean score of physical activity per participant. The mean score per participant was used to represent the physically active lifestyle of the participant and was used to test a between-subjects effect of physical activity on happiness. Also, for each number of steps per day, the deviation in this average number of steps was calculated to represent relative active or inactive days compared to the average behavior of the participant. This relative number of steps of the participant was used to test the within-subjects effect of physical activity on happiness.

#### Happiness

**Fig. 2:**
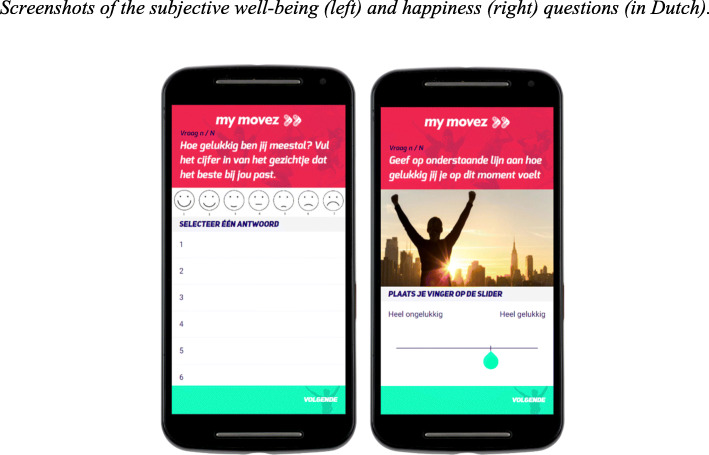
Screenshots of the subjective well-being (left) and happiness (right) questions (in Dutch)

At random moments during the day, participants received an experience sampling question that measured happiness at that specific moment in time: “Indicate on the line below how happy you are at this moment”. Participants responded to the question on a visual analogue scale by placing their finger on a slider ranging from 0 (“very unhappy”) to 100 (“very happy”), see Fig. 2. On average, participants scored rather high on this question (*M* = 75.82, *SD* = 16.52, *Mdn* = 76.24).

In addition, *eudaemonic* well-being was measured once per wave to assess the criterion validity (concurrent validity) of the experience sampling question for happiness. *Eudaemonic* well-being was measured by using the Faces Scale [33], a single-item measure that asks participants “Overall, how do you usually feel?” Participants responded by selecting one of the drawings of faces, arranged in a horizontal line, ranging from 1 (“very happy”) to 7 (“very unhappy”, see Fig. 2). The values were recoded, so an increase in the scale represents an increase in subjective well-being. Again, participants scored on average rather high on the well-being scale (*M* = 5.58, *SD* = 1.09). A Bayesian correlation test (*r* = 0.30, BF_01_ = 57.44 ± 0) indicated very strong evidence for a moderate correlation between the two measures. Therefore, we concluded that *eudaemonic* well-being and the happiness measure were related and deemed that the ESM happiness question was a valid measure of happiness.

#### Covariates

Participants indicated their sex and age at the start of the project. Both were included as covariates because males tend to be more physically active than females, and younger adolescents are more physically active than older adolescents [34]. Also, per day we coded whether this was a week- or a weekend day to control for differences in physical activity [35].

### Strategy of analysis

All data were handled and analyzed in R [36]. To test the hypotheses, Bayesian mixed-effects models were performed by using the BayesFactor package [37], and Jeffreys-Zellner-Siow priors were used. In each analysis, two models were tested against each other: The null model included the dependent variable, the covariates (i.e., sex; age; weekend), and random intercepts per participant and wave and this model was compared to a second model that included the predictor of interest. The outcome of this comparison was the amount of evidence for support for the second model (with the predictor of interest) over the null model (without the predictor of interest), expressed as the Bayes factor. The Bayes factor is the relative strength of evidence for the alternative hypothesis over the null hypothesis. Values greater than 1 indicate evidence for the alternative hypothesis and the higher the value of the Bayes factor, the higher the likelihood of the hypothesis. In contrast, values lower than 1 indicate increasing evidence for the null hypothesis over the alternative hypothesis. When there is no support for either of the two hypotheses, the Bayes factor is close to 1 [38]. For inference of the strength of the support for the hypotheses, the classification of Jeffreys [39] was used.

To test the first set of hypotheses, the day-to-day data were used. The null model of these hypotheses included the sex and age of the participant and the weekend variable as predictors of happiness. In addition, random intercepts per participant and wave were added to the mixed-effects model to account for the clustering of data per participant and wave. Per hypotheses, an alternative model was created by including the predictor of interest. For H1a, the number of steps was included as the predictor. For H1b, the average number of steps per participant was included as the predictor to test the between-subjects effect. For H1c, the number of steps deviating from the mean per participant was included as the predictor to if relative changes in physical activity predict changes in happiness.

To test the second set of hypotheses, a subset of the data was selected which met several criteria. The happiness measure was not missing, the physical activity data of the previous day was not missing, and the physical activity data of the subsequent day was not missing. By default, all happiness measures on the first day and last day of the measurement period were excluded because they did not meet the criteria of having both the previous and subsequent days of physical activity data available. After exclusion, 1673 observations (24.43%) were retained and the subsample consisted of 718 participants (48.61% of the sample). This subsample was slightly younger (*M* = 10.73, *SD* = 1.70) and the percentage of males was lower (42.90%) than in the total sample. The same specifications for the null model and approach were used as in the first set of hypotheses. For H2a, the number of steps on the previous day was used as a predictor of happiness. For H2b, happiness was used as a predictor of the number of steps on the subsequent day. And for H2c, the number of steps on the previous day and happiness were used as predictors of the number of steps on the subsequent day.

To test the third set of hypotheses, the same approach was used as for the previous set of hypotheses, with the modification that the number of steps on the previous and subsequent days was changed to the number of steps during the previous and subsequent hour of the participant’s response to the happiness question. Again, a subset of data was used for which the number of steps during the 5 h surrounding the happiness measures was available. If no steps were recorded during these particular 2 h, the measure was excluded from the analysis. After exclusion, 8498 observations (84.88%) were retained, and the subsample consisted of 1164 participants (78.44% of the participants). Again, the subsample was slightly younger (*M* = 10.73, *SD* = 1.70), but the percentage of males was comparable to the original sample.

## Results

### Preliminary analysis

To replicate the general associations found in previous studies, we first investigated the relationship between the average physical activity and average happiness per participant by calculating the average of both variables per participant. A Bayesian Pearson correlation showed extreme evidence (BF_10_ = 2820.17 ± 0%) for a weak correlation (*r* = 0.13, *SE* = 0.00) between the average physical activity and average happiness per participant in the project. This corroborates previous findings and indicated that indeed physical activity and happiness in adolescents were associated.

### Hypothesis 1

**Fig. 3:**
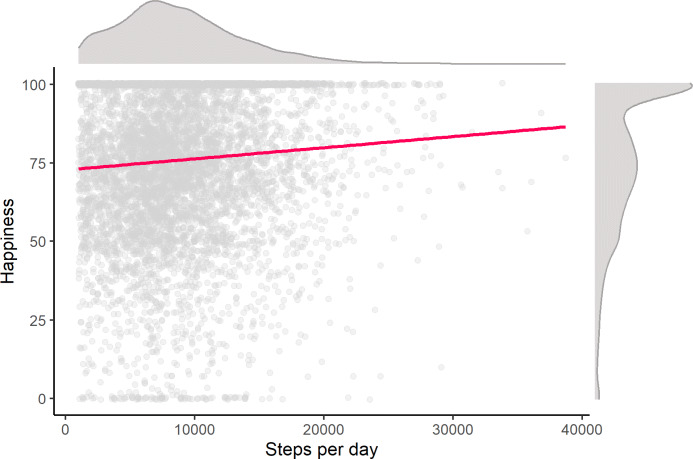
The association between the number of steps per day and happiness in adolescents. Note. Grey dots are the observed values, the red line is the estimated linear coefficient

The first set of hypotheses investigated the association between the daily number of steps and the reported happiness on that day. To test H1a, a Bayes mixed-effect model tested the amount of evidence that the number of steps predicted the experience sampled happiness per day. In line with our expectations, the data were many times more likely under the alternative model than the null model (BF_10_ = 30.60 ± 0.04%). This means that there was strong evidence that an increase of 1000 steps per day was associated with an increase of 0.20 (*SE* = .001) in the reported happiness on that day. Figure 3 represents the association between the daily number of steps and happiness per participant.

To test H1b, the average number of steps per participant was used to test the between-subjects effects of physical activity on happiness. In other words, we wanted to test whether participants who are on average more physically active are happier. In line with our expectations, the data were more likely under the alternative model than the null model (BF_10_ = 4.90 ± 0.04%). This means that there was moderate evidence that when adolescents were on average 1000 steps per day more active, they reported 0.46 (*SE* = .002) points higher on the happiness scale. Therefore, we can conclude that adolescents who are more physically active in general are happier than those who are less physically active.

To test H1c, the variable that measured the deviations in the participants’ average physical activity was used to test the within-subjects effects of physical activity on happiness. In other words, we wanted to test whether participants were happier when they were relatively more physically active on that specific day than their average behavior. The data were only slightly more likely under the alternative model than the null model (BF_10_ = 1.75 ± 0.05%). This means that there was only anecdotal evidence that participants were happier when they were relatively more physically active. Per 1000 steps increase compared to their average number of steps, adolescents scored only a little higher on the happiness questionnaire (*B* = 0.16, *SE* = .001). This means that we are less certain about the evidence for the within-subjects effect, and the effect is relatively smaller than the between-subjects effect of physical activity on happiness.

### Hypothesis 2

The second set of hypotheses investigated the direction of the association between physical activity and happiness in adolescents. More specifically, these analyses tested whether physical activity on the previous day predicted changes in happiness and whether happiness predicted changes in physical activity on the subsequent day. To test H2a, a Bayes mixed-effect model tested the amount of evidence that the number of steps of the previous day predicted happiness on that day. Contrary to our expectations, the data were more likely under the null model than the alternative model (BF_10_ = 0.09 ± 0.08%), which qualifies as very strong evidence that there was no effect from the number of steps of the previous day on happiness in adolescents.

To test H2b, a similar model tested the amount of evidence that happiness predicted changes in the number of steps in the subsequent day. The data were equally likely under the alternative model than the null model (BF_10_ = 0.99 ± 0.05%). A Bayes factor close to 1 indicates that there is inconclusive evidence for the effect of happiness on the number of steps on a subsequent day.

To test H2c, the mediating role of happiness in the autoregressive effect of physical activity was investigated. To test this mediation, the approach as described in Nuijten et al. [40] was used. Very strong evidence was found that there was no indirect effect of the number of steps during the previous day on the number of steps on the subsequent day that was mediated by happiness (BF_10_ = 0.04),. In contrast, the data showed extreme evidence for the alternative model that included the autoregressive effect of physical activity 2 days later over the null model (BF_10_ = 4.68*10^9^ ± 0.03%). Therefore, happiness does not mediate physical activity on a day-to-day level, and we reject hypothesis 2c.

### Hypothesis 3

The third set of hypotheses investigated the direction of the association between physical activity and happiness in adolescents within the time frame of an hour. In these analyses, we tested whether happiness was predicted by the amount of physical activity during the previous hour and whether happiness predicts physical activity in the subsequent hour. The same approach was used for the previous set of hypotheses, but now the number of steps of the previous and subsequent hours were used to assess a more immediate association between physical activity and happiness.

To test H3a, a Bayes mixed-effect model tested the amount of evidence that the number of steps during the previous hour predicted happiness. In line with our expectations, the data were many times more likely under the alternative model than the null model (BF_10_ = 143.13 ± 0.03%), which qualifies as very strong evidence that the number of steps of the previous hour predicted happiness in adolescents. Per increase of a thousand steps per hour, participants scored 0.66 (*SE* = .002) higher on the happiness scale.

To test H3b, a similar model tested the amount of evidence that happiness predicted the number of steps in the subsequent hour. The data were more likely under the alternative model than the null model (BF_10_ = 1962.89 ± 0.14%), which qualifies as extreme evidence that happiness predicted the number of steps in the subsequent hour. Per increase in the happiness variable, participants increased by 1.01 (*SE* = .002) steps in the subsequent hour.

To test H3c, the mediating role of happiness on an hour-to-hour level, the same approach as before was used [40]. First, the indirect effect was assessed. The data were more likely under the alternative model than the null model (BF_10_ = 136.92), which qualifies as very strong evidence that there was an indirect effect of the number of steps during the previous hour on the number of steps during the subsequent hour, that was mediated by happiness. In addition, the data showed extreme evidence for the alternative model that included the autoregressive effect of physical activity 2 h later over the null model (BF_10_ = 1.02 × 10^83^± 0.38%). Therefore, we can conclude that happiness partially, but marginally, mediated physical activity on an hour-to-hour level and we can confirm hypothesis 3c.

## Discussion

This study aimed to extend the current knowledge about the association between physical activity and happiness in adolescents and to determine the directionality. In a series of analyses, we have demonstrated that physical activity and happiness were indeed related to one another. More specifically, the analyses showed that happiness sampled at random moments during the day was predicted by the participant’s number of steps on that day. Moreover, the results show that living a physically active lifestyle was associated with greater happiness in general, but only anecdotal evidence (according to the evidence categories by Jeffreys [39]) was found that relative deviation from the average physical activity of the participant was associated with changes in happiness.

In addition, this study showed a reciprocal short-term effect of physical activity and happiness in adolescents. More specifically, when adolescents are more physically active, they feel happier, and when they feel happy, they also became more physically active in the subsequent hour. On the contrary, this reciprocal relationship was not observed between days. Therefore, we conclude that physical activity and happiness in adolescents only have a short-term (hour-to-hour) reciprocal effect on each other.

The observed general associations between physical activity and happiness are in line with cross-sectional studies that have observed positive associations between self-reported physical activity and mental well-being [10, 11] or happiness [12–14]. The current study extends this body of knowledge by showing that the evidence is most convincing (i.e., highest Bayes factor) when accounting for the day-to-day fluctuations in physical activity and happiness, more so than looking at the average physical activity of the participant. However, our findings show that this cannot be explained by the variation in physical activity on the part of the participant, as we have found more convincing evidence that the average levels of physical activity predicted changes in happiness rather than the relative deviations from this average. From this finding, we can infer that living a physically active lifestyle contributes to an increase in happiness in adolescents, and deviations from the average lifestyle only have a marginal effect on the experienced happiness on that day.

Furthermore, our finding regarding the short-term reciprocal effect could indicate a vicious cycle in which physical activity makes adolescents feel happier, which will then make them more physically active again. Given that this study did not observe this effect between days is an explanation as to why the previous longitudinal study [29] did not find an effect of physical activity on happiness, as it could be that their time frame of 3 years is too large. The observed time frame of an hour in this study is also more in line with the physiological processes that provide an explanation of why adolescents become happier after being physically active, as these processes are measurable between 30 min to an hour after the activity [17, 41]. This short time frame also gives an explanation of why only anecdotal evidence was found for the within-subject effects of physical activity among adolescents. Potentially these deviations among adolescents only have a short-term effect and do not hold for the entire day.

These findings implicate that the current behavioral theories (e.g., theory of planned behavior [18], self-determination theory [19], the trans-theoretical model [21]) are limited in the fact that they only predict changes in physical activity as a result of changes in happiness. Moreover, these results echo the call by Ekkekakis et al. [22] to develop theoretical models of physical activity that acknowledge the important role of affect in behavioral decision-making. However, these results show that physical activity is not the definitive step in the chain of events and that the theoretical models could be improved by acknowledging a backward loop from behavior to, for example, beliefs or affective states. In addition, these findings are valuable to those who aim to increase the health of adolescents by targeting their health-related behaviors. It can be difficult to convince youth of the importance of sufficient physical activity, and the benefits of a healthy lifestyle can be hard to comprehend for the target audience. Focusing the message on happiness can increase the effectiveness of interventions because this relates more to the motivational drivers for their behavior and can make the intervention message more relevant and persuasive to them.

### Strengths and limitations

By using ESM to measure happiness, we were able to address some of the shortcomings of retrospective self-reported measures of happiness. In addition, with the continuous objectively measured physical activity, we were able to further investigate the association between physical activity and happiness in adolescents and strengthen the knowledge about the reciprocal effects they have on each other. Furthermore, this study has pursued open-science practices in which publicly available data has been used and the hypotheses and analyses have been preregistered. This transparency and using Bayesian hypothesis testing strengthens our confidence in these findings.

Future research should further investigate the time frame in which physical activity and happiness affect each other. In the design used, the physical activity of the previous and subsequent hour were chosen because this was in line with the hypothesized physiological mechanism and the smallest time frame possible in the project data. However, it is plausible that happiness has even a more immediate effect on physical activity (e.g., in a matter of seconds). In addition, other types of measures could be used by, for example, using the heart-rate of the participant, directly after answering the ESM happiness questionnaire as a proxy for physical activity at that time. Heart-rate measures were not included in the project, and therefore could not be used in the current study. Potentially, the more immediate measures of physical activity will result in even more evidence or stronger effects of the happiness measurement and vice versa.

In the project, experience sampling methodology was used in which the participants received a visual analogue scale at random time points during the day. Participants scored on average relatively high on this measure, and some participants indicated the maximum score (100) on the happiness question. On the one hand, this relatively high level of happiness and well-being have been demonstrated more often in Dutch adolescents [33, 42]. On the other hand, sensitivity analyses have indicated that removing extreme scores on both measures could result in higher estimated association between the two variables and higher Bayes factors. More variation in the question could potentially be achieved by measuring happiness with more than one item or changing the labels of the extreme values to give a broader perspective to the visual analogue scale. For example, the current labels (“very unhappy” and “very happy”) could be changed to “worse than usual” and “better than usual”. Probably, this will reduce the number of times that participants indicate extreme values on the scale.

A third limitation of the study is that the number of steps is as a measure of physical activity. The used accelerometer could not reliably measure sleep or sedentary activities, which both could have its distinctive positive and negative contribution to mental well-being. Also physical activity might only be a part of the health related-behaviors that influence mental well-being. For example, a more complete assessment of healthy lifestyles could elucidate what factors increase happiness in adolescents. For example, future studies could include measures of sleep, dietary intake, or sedentary behaviors to extend the knowledge about the relationship between a healthy lifestyle and happiness. However, it is important to recognize that these health-related behaviors are not occurring on such a micro-level as physical activity. That is, most people only sleep once per 24 h and only consume a limited number of meals per day. Therefore, a broader healthy lifestyle perspective would not fit the micro-level perspective that is used in the study.

## Conclusions

This study was the first to investigate the relationship between physical activity and happiness in adolescents, utilizing accelerometers to continuously measure physical activity and an experience sampling methodology to measure happiness at random moments during the day. Our findings have convincingly shown that physical activity is associated with happiness in adolescents and that there is a short-term reciprocal effect of physical activity on happiness from 1 h to the next, but not between days. On the one hand, these findings implicate that current behavioral theories should acknowledge that physical activity is not a definitive outcome, but part of an iterative process. On the other hand, the findings illustrate one of the many benefits of physical activity in youth. Therefore, interventions promoting physical activity in adolescents can also argue that physical activity is not only beneficial for youth’s health but will also increase their happiness.

## Data Availability

The data used in this publication are stored at the Data Archiving and Networked Services (10.17026/dans-zz9-gn44). Hypotheses, study design, sample, data collection procedure, measured variables, and plan of analysis were preregistered on the Open Science Framework (OSF, https://osf.io/5yk7r).
